# Impact of Endobronchial Ultrasound-Guided Transbronchial Needle Aspiration (EBUS-TBNA) on Lung Carcinoma Staging: A Retrospective Study

**DOI:** 10.7759/cureus.17963

**Published:** 2021-09-14

**Authors:** Konstantinos Kosmas, Andreas Kosmas, Dimitra Riga, Christos Kyritsis, Nefeli Georgia Riga, Evangelos Tsiambas

**Affiliations:** 1 Department of Cytopathology, 417 Army Equity Fund Hospital (NIMTS), Athens, GRC; 2 2nd Intensive Care Unit, General Hospital of Thessaloniki “George Papanikolaou”, Exohi, Thessaloniki, GRC; 3 Pathology Department, General Hospital of Thoracic Diseases of Athens “Sotiria”, Athens, GRC; 4 Intensive Care Unit, General Hospital of Thoracic Diseases of Athens “Sotiria”, Athens, GRC

**Keywords:** lung cancer, mediastinal lymph node, cytology, ebus-tbna, endobronchial ultrasound-guided transbronchial needle aspiration

## Abstract

Introduction: Lung cancer is the most common cancer in the world, both in terms of new cases and deaths. Almost a fifth of all cancer deaths worldwide are due to lung cancers. Our aim was to evaluate the utility of endobronchial ultrasound-guided trans-bronchial needle aspiration (EBUS-TBNA) for lymph node staging in patients with lung cancer.

Methods: We performed a retrospective study on a total of 427 patients who underwent EBUS-TBNA sampling from January 2020 to December 2020 and a total of 610 lymph nodes were sampled. There were 322 men (mean age: 66.3 and range: 20-87) and 105 women (mean age: 65.9 and range: 18-81).

Results: Cytological diagnosis revealed that 55 patients had adenocarcinoma, 28 squamous cell carcinoma, 43 neuroendocrine tumours, 34 non-small cell carcinoma not otherwise specified, 21 metastasis from extra-thoracic malignancy, 7 atypical cells suspicious for malignancy, and 239 patients had normal or reactive lymph nodes or non-neoplastic diagnosis. The diagnostic accuracy, sensitivity, specificity, positive predictive value (PPV) and negative predictive value (NPV) were 91%, 88.3%, 100%, 100% and 89.2%, respectively.

Conclusion: EBUS-TBNA is a safe technique with high accuracy, sensitivity, specificity, PPV, and NPV. It is an excellent option for the diagnostic approach of patients with lymphadenopathy or intra-thoracic lesions as well as for the staging of malignancies.

## Introduction

Lung cancer is the most common cancer in the world, both in terms of new cases (1.8 million) and deaths (1.6 million) per year. Almost a fifth of all cancer deaths worldwide are due to lung cancers (1.59 million per year) [1]. 

Endobronchial ultrasound-guided trans-bronchial needle aspiration (EBUS-TBNA) has emerged as a novel, minimally invasive procedure for the diagnosis of lymphadenopathies (mediastinal and hilar lymph nodes) and/or mediastinal masses of different etiologies, as well as for staging of pulmonary and extra-pulmonary neoplasms [2,3]. Its main advantages include improved accessibility of the lesions because of real-time puncturing, with direct observation of lesions and regional vessels, thus increasing the chances of adequate specimen collection and minimizing the risk of significant bleeding [3].

In the hands of an experienced operator, the procedure is safe, cost-effective, provides excellent sensitivity, specificity, and predictive diagnostic values as well as excellent cytologic specimens that have proven well suited for ancillary testing, such as immunohistochemistry and tumor genotyping [4]. Our aim in this study was to evaluate the utility of EBUS-TBNA for lymph node staging in patients with lung cancer.

## Materials and methods

We performed a retrospective study based on a total of 427 patients who underwent EBUS-TBNA sampling from January 2020 to December 2020 in various institutions in Greece specialized in chest diseases. A total of 610 lymph nodes were sampled with an average of 1.43 lymph nodes per patient. There were 322 men (mean age: 66.3 and range: 20-87) and 105 women (mean age: 65.9 and range: 18-81). All the patients tolerated the procedure quite well, without major complications related to EBUS-TBNA, except minimal self-limited wound oozing during the procedure. The aim of EBUS-TBNA was to establish the diagnosis of an enlarged lymph node of unknown cause or to accurately stage patients with lung cancer. The procedure can also be used for the diagnosis of inflammatory and infectious conditions. Before the examination, all patients underwent a clinical evaluation with a chest computed tomography (CT) scan and/or positron emission tomography/computed tomography (PET/CT) for the planning of the procedure. The procedures were performed by pulmonologists and/or thoracic surgeons, who were skilled and experienced in the method.

The slides were fixed in 95% ethanol and stained with the Papanicolaou technique (MERCK® KGaA, Darmstadt, Germany) following the manufacturer's protocol. For immunocytochemistry, smears were air-dried and ﬁxed in ethanol/acetone 1:1 for 10 minutes and stored at -35°C until used. Immunostaining was performed by the MENARINI Bond Max (A. MENARINI Diagnostics, Athens, Greece). Non-diagnostic samples included mucus, blood clots, necrotic tissue, in which we were not able to confirm adequate cell material and looked for the cells of interest. The origin of malignant cells was detected either morphologically alone on the basis of malignant cytological results at EBUS-TBNA specimen or in combination with the use of immunocytochemical markers (TTF-1, Ber-EP4, CD56, p63, CK7, CK20, etc.) if needed.

The samples that were normal or benign (even if lymphadenopathies were considered highly suspicious for recurrence based on CT and/or PET scan characteristics), were classified as “negative”. The aspirate samples from the lymph node sites were categorized as “atypical cells suspicious for malignancy” when dysplastic bronchial epithelium was present or when rare cells suspicious for malignancy were seen. In our study, only cases with cytologic diagnosis correlated and confirmed with available transbronchial biopsy or surgical resection specimen were included. In case the diagnostic EBUS-TBNA sample was normal, but it was classified as malignant after the confirmatory procedure, the result was classified as “false-negative” and if the EBUS-TBNA sample was negative for malignancy, but it was considered normal after the confirmatory procedure, the result was defined as “false-positive”.

EBUS-TBNA Technical aspects

A convex probe endobronchial ultrasound (CP-EBUS; BF-UC180F, Olympus, Tokyo, Japan) was used for EBUS-TBNA. The ultrasound image was processed with a universal endoscopic ultrasound scanner (EU-ME1; Olympus, Tokyo, Japan) and once the target lymph node station was identified (by ultrasound), a dedicated 21-gauge needle (Vizishot NA-201SX-4021; Olympus, Tokyo, Japan) was inserted into the working channel to perform real-time TBNA. 

Statistical analysis

Statistics software package SPSS version 25 (IBM Corp., Armonk, NY) was implemented. The sensitivity, specificity, positive predictive value (PPV), negative predictive value (NPV), and diagnostic accuracy rate were calculated via standard definitions.

## Results

Cytological diagnoses, patient characteristics, and the number of sampled lymph nodes are reported in Table 1. In 181 patients, metastatic involvement of the lymph node was established cytologicaly and confirmed by histological examination. In 239 patients with negative diagnosis for malignancy of EBUS-TBNA, the false-negative cases revealed by pathologic examination were 22 (9.2%). In the present study, the diagnostic accuracy, sensitivity, specificity, PPV and NPV were 91%, 88.3%, 100%, 100% and 89.2%, respectively. No false-positive case was encountered.

**Table 1:** Cytological diagnoses, patient characteristics, and number of sampled lymph nodes NSCLC - Non‑small cell lung cancer; SCC - Squamous cell carcinoma; SCLC - Small cell lung carcinoma; NSCLC-NOS - Non‑small cell lung cancer-not otherwise specified; LN - Lymph node

Cytological diagnosis	No. of Patients	Gender	Mean age	Range	No. of LNs
NSCLC-Adenocarcinoma	55	Male:34 Female:21	67 70	30-80 50-78	78
NSCLC-SCC	28	Male:23 Female:5	68 66.5	57-83 63-74	40
Neuroendocrine tumour (Including SCLC & carcinoid)	43	Male:37 Female:6	67.7 70.5	48-78 54-81	62
NSCLC-NOS	34	Male:27 Female:7	64 60	42-84 42-78	50
Metastasis from extrathoracic malignancy	21	Male:17 Female:4	65.6 64.6	23-79 18-68	28
Atypical cells suspicious for malignancy	7	Male:6 Female:1	65.2 61	54-78 61	10
Normal or reactive LNs, non neoplastic diagnosis	239	Male:177 Female:62	65.6 64	20-87 24-80	342

**Figure 1:**
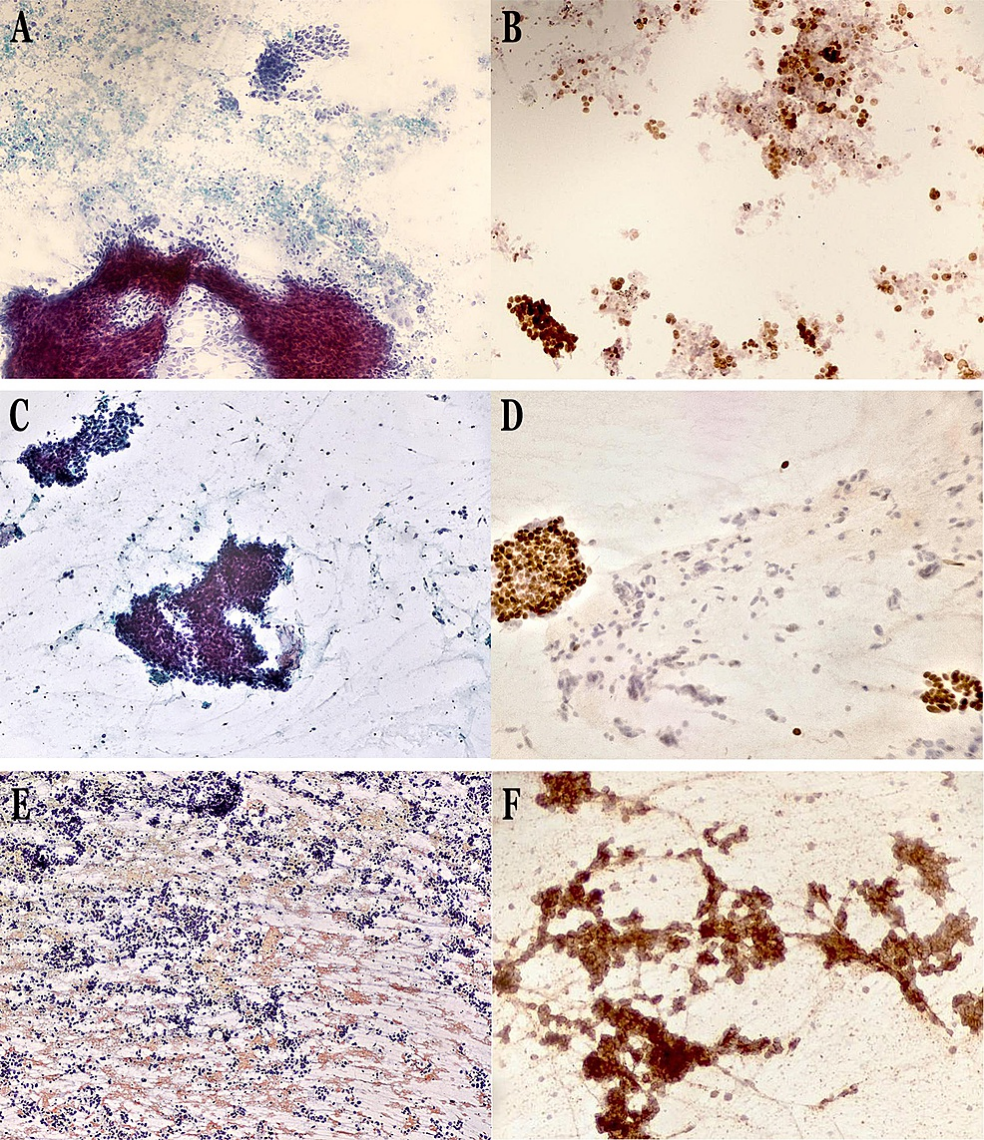
Representative pictures of EBUS-TBNA based cytological samples. A. Adenocarcinoma (Papanicolaou stain, x200). B. Adenocarcinoma (TTF-1 positive staining, x200). C. Squamous cell carcinoma (Papanicolaou stain, x200). D. Squamous cell carcinoma (p63 positive staining, x200). E. Small cell lung carcinoma (Papanicolaou stain, x100). F. Small cell lung carcinoma (CD56 positive staining, x400).

Figure 1 provides representative pictures of cytological specimens. 

## Discussion

Over the past two decades, EBUS-TBNA has emerged as a highly effective and minimally invasive technique for sampling peribronchial, mediastinal, and lung masses for cytopathologic examination [4]. EBUS-TBNA in patients with suspected lung cancer in many instances has become the first-line approach for cytopathologic diagnosis and staging, due to its excellent specificity, positive and negative predictive values, good sensitivity, and high diagnostic accuracy [5], partly attributed to the expertise and skill of the bronchoscopist performing the procedure as well as the cytopathologist interpreting the specimen [4,6]. A summary of the diagnostic performance of EBUS in a collection of recent and large-scale studies is presented in Table 2. In our study, the diagnostic accuracy, sensitivity, specificity, PPV and NPV were 91%, 88.3%, 100%, 100% and 89.2%, respectively.

**Table 2:** Summary of diagnostic performance of EBUS in a collection of recent and large-scale studies EBUS - Endobronchial Ultrasound; PPV - Positive predictive value; NPV - Negative predictive value

	No. of Patients	Accuracy %	Sensitivity %	Specificity %	PPV %	NPV %
Aljohaney AA [5]	52	79.0	78.6	100	100	80
Murthi et al. [7]	139	81.2	55.1	100	100	75.7
Zhang et al. [8]	55	83.6	65.2	96.9	93.8	79.5
Guarize et al. [9]	1891	93.6	91.7	100	100	78.5
Um et al. [10]	138	92.9	88.0	100	100	85.2

In the present study, false-negative EBUS-TBNA results for mediastinal staging were observed in 9.2% of patients. However, some studies have demonstrated that EBUS-TBNA has a significantly high false-negative rate for diagnosis and staging of thoracic malignancy [7,11,12] as high as 24% [13] due to intranodal necrosis, rare types of malignancy, the inadequacy of the EBUS-TBNA specimens [14], large number of EBUS-TBNA accessible lymph nodes that are not sampled [7], as well as the extensiveness of sampling or lesions out of reach of EBUS-TBNA, particularly in left-sided tumours [11].

Some literature studies have described the utility of EBUS-TBNA in other pathologies with mediastinal lymphadenopathies such as tuberculosis with an overall accuracy rate of 91%, sensitivity of 62%, specificity of 100% and NPV of 89%, respectively [15], lymphoma with an overall accuracy rate of 96% [16], sensitivity ranging from 90.9% [16] to 76% [17], specificity of 100% [16,17], PPV of 100% [16] and NPV ranging from 87% [17] to 92,6% [16], respectively and sarcoidosis [18] with diagnostic accuracy ranging from 85% [19] to 87.5% [20] and sensitivity of 85% [19].

World Health Organization divides lung cancer into two ma­jor classes based on its biology, therapy, and prog­nosis: Non-small cell lung cancer (NSCLC) and small cell lung cancer (SCLC) [21]. The need to further subcategorize NSCLC into adenocarcinoma versus squamous cell carcinoma is crucial because of implications for the selection of appropriate chemotherapy as well as identifying cases that should be tested for the presence of a driver mutation and can be specifically treated with a targeted agent [4].

EBUS-TBNA samples are now being routinely used for molecular analyses [22]. Several biomarkers have emerged as predictive and prognostic markers for NSCLC. A predictive bio­marker [the ALK fusion oncogene, a fusion between *ALK* (anaplastic lymphoma receptor tyrosine kinase) and other genes e.g., echinoderm microtubule-associated protein-like 4 (*EML4*); sensitizing epidermal growth factor receptor (*EGFR*) mutations; proto-oncogene 1, receptor tyrosine kinase (*ROS1*) gene rearrangements; programmed death-ligand 1 (PD-L1); Kirsten rat sarcoma viral oncogene homolog (*KRAS*)] is indicative of therapeutic efficacy because there is an interaction between the biomarker and therapy on patient outcome. A prognostic biomarker (*HER2*; *BRAF* V600E mu­tations; *RET* gene rearrangements; high-level MET amplification) is indicative of patient survival independent of the treatment received because the biomarker is an indicator of the innate tumor aggressiveness [23].

Moreover, EBUS-TBNA has an excellent safety profile [2,4,5,19,20,24,25] in the hands of an experienced operator and it is safer than surgical mediastinoscopy, a complimentary staging modality that has been considered the standard procedure for staging [4]. Its potential complications, although rare, include pneumothorax, mediastinitis, pneumomediastinum, bacteremia, and hemomediastinum [4]. Large series have produced a major complication rate of approximately 0.15% and only two deaths have been reported in the literature [25-27]. It has been proven a safe technique even in elderly patients as Guarize et al. reported in their study (732 out of 1891 patients over 70 years old and 120 patients over 80 years old) [9].

## Conclusions

Conclusively, our results refer to a retrospective study performed at high-volume thoracic institutions in Greece, specialized in chest diseases, with procedures carried out by many experienced bronchoscopists working with high-volume of samples received in the department of cytopathology. Based on our experience, we observed that this minimally invasive outpatient procedure is safe with high accuracy, sensitivity, specificity, PPV, and NPV. EBUS-TBNA is an excellent option for the diagnostic approach of patients with lymphadenopathy or intra-thoracic lesions as well as for the staging of neoplasms, and it is also an ideal procedure to provide adequate tumor specimens for genetic assessment in advanced lung cancer patients.
